# PP2A inhibitors arrest G2/M transition through JNK/Sp1-dependent down-regulation of CDK1 and autophagy-dependent up-regulation of p21

**DOI:** 10.18632/oncotarget.4063

**Published:** 2015-05-25

**Authors:** Fei-Ran Gong, Meng-Yao Wu, Meng Shen, Qiaoming Zhi, Ze-Kuan Xu, Rong Wang, Wen-Jie Wang, Yang Zong, Zeng-Liang Li, Yadi Wu, Binhua P. Zhou, Kai Chen, Min Tao, Wei Li

**Affiliations:** ^1^ Departments of Oncology, the First Affiliated Hospital of Soochow University, Suzhou, China; ^2^ Departments of Hematology, the First Affiliated Hospital of Soochow University, Suzhou, China; ^3^ Jiangsu Institute of Hematology, the First Affiliated Hospital of Soochow University, Suzhou, China; ^4^ Key Laboratory of Thrombosis and Hemostasis of Ministry of Health, the First Affiliated Hospital of Soochow University, Suzhou, China; ^5^ Collaborative Innovation Center of Hematology, Soochow University, Suzhou, China; ^6^ Department of General Surgery, the First Affiliated Hospital of Soochow University, Suzhou, China; ^7^ Department of General Surgery, the First Affiliated Hospital of Nanjing Medical University, Nanjing, China; ^8^ Department of General Surgery, the Changshu No.1 People's Hospital, Changshu, China; ^9^ Departments of Molecular and Biomedical Pharmacology, University of Kentucky College of Medicine, Lexington, KY, USA; ^10^ Departments of Molecular and Cellular Biochemistry, University of Kentucky College of Medicine, Lexington, KY, USA; ^11^ Markey Cancer Center, University of Kentucky College of Medicine, Lexington, KY, USA; ^12^ Jiangsu Institute of Clinical Immunology, Suzhou, China; ^13^ Institute of Medical Biotechnology, Soochow University, Suzhou, China; ^14^ PREMED Key Laboratory for Precision Medicine, Soochow University, Suzhou, China

**Keywords:** PP2A, G2/M cell cycle arrest, JNK, CDK1, Sp1

## Abstract

Protein phosphatase 2A (PP2A) plays an important role in the control of the cell cycle. We previously reported that the PP2A inhibitors, cantharidin and okadaic acid (OA), efficiently repressed the growth of cancer cells. In the present study, we found that PP2A inhibitors arrested the cell cycle at the G2 phase through a mechanism that was dependent on the JNK pathway. Microarrays further showed that PP2A inhibitors induced expression changes in multiple genes that participate in cell cycle transition. To verify whether these expression changes were executed in a PP2A-dependent manner, we targeted the PP2A catalytic subunit (PP2Ac) using siRNA and evaluated gene expression with a microarray. After the cross comparison of these microarray data, we identified that CDK1 was potentially the same target when treated with either PP2A inhibitors or PP2Ac siRNA. In addition, we found that the down-regulation of CDK1 occurred in a JNK-dependent manner. Luciferase reporter gene assays demonstrated that repression of the transcription of CDK1 was executed through the JNK-dependent activation of the Sp1 transcription factor. By constructing deletion mutants of the CDK1 promoter and by using ChIP assays, we identified an element in the CDK1 promoter that responded to the JNK/Sp1 pathway after stimulation with PP2A inhibitors. Cantharidin and OA also up-regulated the expression of p21, an inhibitor of CDK1, via autophagy rather than PP2A/JNK pathway. Thus, this present study found that the PP2A/JNK/Sp1/CDK1 pathway and the autophagy/p21 pathway participated in G2/M cell cycle arrest triggered by PP2A inhibitors.

## INTRODUCTION

Natural products and their derivatives have shown promising outcomes in cancer therapies. In our previous study, we demonstrated that cantharidin, the active constituent of Chinese blister beetle, [1] inhibited growth of pancreatic and breast cancer cells, [2, 3] offering a potential role for cantharidin in cancer treatment. Cantharidin acts as a potent and selective inhibitor of protein phosphatase 2A (PP2A), [4] a multimeric serine/threonine phosphatase that is generally considered to be a cancer repressor. On one hand, inhibition of PP2A is believed to have tumor-promoting function [5] through induction of phosphorylation and activation of several substrate kinases, including IKK (IκB kinase), JNK (c-Jun N-terminal kinase), ERK (extracellular signal-related kinase), p38, Akt, and PKC (protein kinase C), most of which accelerate growth. [6, 7] On the other hand, recent studies showed that PP2A inhibition by specific inhibitors also elicit growth-suppressive effect through several growth inhibition pathways, [8, 9] including the JNK pathway that we reported previously. [2] Inhibition of this pathway significantly reduced the cytotoxic effect of cantharidin and okadaic acid (OA; another classic PP2A inhibitor), [2] suggesting that inhibition of PP2A indeed has tumor suppressive function. Thus, understanding the molecular mechanism underlying the growth-suppressive effect of PP2A inhibition will not only minimize the unwanted side effect of PP2A inhibition but also facilitate the development of PP2A inhibitors (i.e., cantharidin) as new therapeutic agents for cancer treatment.

In our previous study, we found that treatment with PP2A inhibitors arrested G2/M cell cycle transition. [2] However, it remained unknown whether G2/M cell cycle arrest was induced through the JNK pathway dependent manner, and whether G2/M arrest is sufficient to suppress the growth of cancer cells. In the present study, we dissected the detailed signaling mechanisms involved in the G2/M cell cycle arrest and JNK dependent cell-killing effect induced by PP2A inhibitors in pancreatic, breast and lung cancer cells.

## RESULTS

### PP2A inhibitors repressed G2/M cell cycle transition

**Figure 1:**
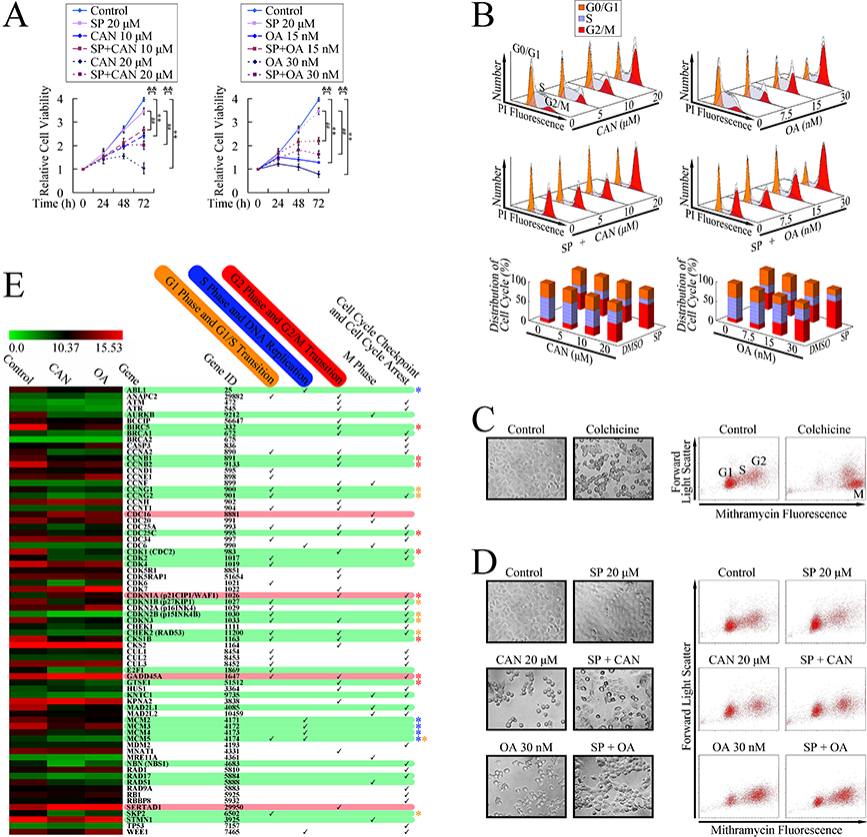
G2/M cell cycle arrest in PANC-1 cells after PP2A inhibitor treatment **A.** Growth curve of cantharidin (CAN), and okadaic acid (OA) treated cells, with or without the pretreatment with JNK inhibitor, SP600125 (SP). **P < 0.01 vs. respective control groups; ^##^*P* < 0.01 vs. SP600125 group; ^&&^*P* < 0.01 induction between groups. **B.** DNA histogram and cell cycle distribution after treatment with cantharidin and OA, pretreated with or without SP600125. **C.** Photomicrographs of Colchicine treated cells (×40); and flow cytometry analyses based on mithramycin A staining. **D.** Photomicrographs of PP2A inhibitors and JNK inhibitor-treated cells (×40); and flow cytometry analyses based on mithramycin A staining. **E.** Illustration of the microarrays results. Up-regulated genes were highlighted in red below, and down-regulated genes were highlighted in green below. The differences in expression changes reached more than 2-fold are marked with colored * (yellow, genes involved in the regulation of G1 phase and G1/S cell cycle transition; blue, genes involved in the regulation of S phase; red, genes involved in the regulation of G2 phase and G2/M cell cycle transition).

Dose- and time-dependent repression on cell growth by PP2A inhibitors was firstly confirmed by MTT assay. Pretreatment with SP600125, the classic JNK inhibitor, attenuated the growth repression by PP2A inhibitors, indicating the JNK pathway dependent cytotoxicity of PP2A inhibitors (Figure 1A and Supplementary Figure 1). In order to investigate whether G2/M cell cycle arrest was also executed through the activation of JNK pathway, we used flow cytometry, based on PI staining, to analyze the cell cycle distribution. As shown in Figure 1B, PP2A inhibitors induced cell accumulation at G2/M phase in a dose-dependent manner, with concurrent decreases in cell populations at G0/G1 and S phases, after 24 h treatment. The level of G2/M aggregation was reduced by pretreatment with SP600125, suggesting that PP2A inhibitors induced G2/M arrest through a JNK dependent pathway.

As flow cytometry analysis based on PI staining cannot distinguish between G2 and M cell cycle arrest, because cells at both these phases are diploid; flow cytometry analysis based on mithramycin A staining was performed. [10] Colchicine, an established M phase blocker, was used as the positive control for inducing M phase cell cycle arrest. As shown in Figure 1C, colchicine treated cells became round and detached; and flow cytometry analysis, based on mithramycin A staining, confirmed that the cells accumulated in M phase. Similarly, cells treated with PP2A inhibitors also became round and detached (Figure 1D). Furthermore, pretreatment with the JNK inhibitor, SP600125, protected cells from this morphological change; however, neither of these treatments induced accumulation of cells in M phase (Figure 1D), which suggested that G2/M cell cycle arrest induced by PP2A inhibitors occurred at G2 phase.

The cell cycle transition is regulated by cyclin, cyclin-dependent kinases (CDKs), and cyclin-dependent kinase inhibitors (CKIs). [11, 12] To investigate which genes were involved in the cell cycle alteration triggered by PP2A inhibitors, we performed microarray analyses to determine the mRNA expressions of 72 genes participating in cell cycle regulation. Among these genes, expression changes of 21 genes consist with the decreases in cell populations at G0/G1 and S phases, as well as arrested G2/M transition in both cantharidin and OA treated groups (Figure 1E), suggesting multiple genes were involved in the cell cycle regulation by PP2A inhibitors.

### PP2A inhibitors repressed cell growth through JNK dependent downregulation of CDK1

**Figure 2:**
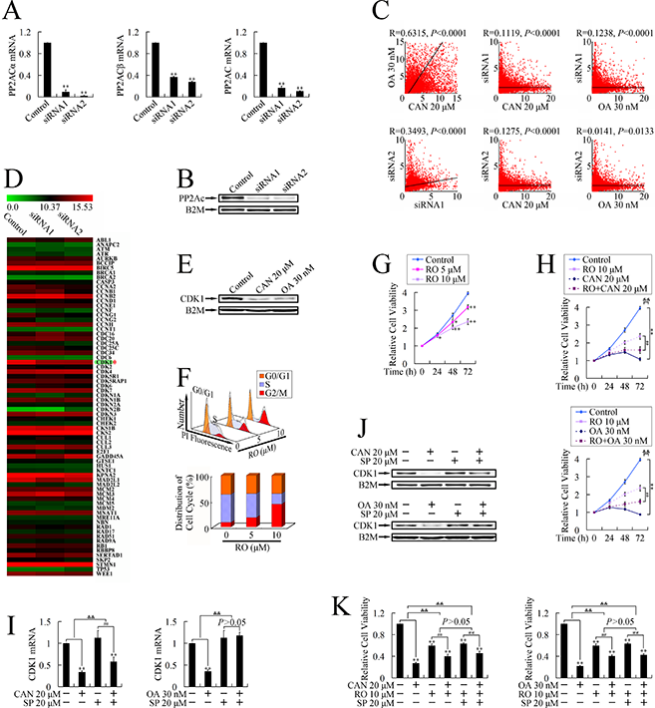
Identification of CDK1 as the target gene at the downstream of PP2A/JNK pathway upon treatment with PP2A inhibitors in PANC-1 cells **A.** and **B.** Confirmation of knockdown of PP2Ac by using real-time PCR (A) and western blot (B). **C.** Pairwise comparison of gene expression profiles upon treatments with cantharidin (CAN), OA and PP2Acα siRNAs by using spearman's rank correlation analysis. **D.** Illustration of the microarrays results. **E.** Confirmation of down-regulated CDK1 upon treatment with PP2A inhibitors by using western blot. **F.** DNA histogram and cell cycle distribution after treatment with RO-3306. **G.** Growth curve of RO-3306 treated cells. *P < 0.05, **P < 0.01 vs. control group. **H.** Growth curve of cantharidin, and OA treated PANC-1 cells, with or without the pretreatment with RO-3306. **P < 0.01 vs. respective control groups; ^##^*P* < 0.01 vs. RO-3306 group; ^&&^*P* < 0.01 induction between groups. **I.** and **J.** After pretreatment with the JNK inhibitor, SP600125, for 3 h, PP2A inhibitors were added into the culture, and cells were treated for 24 h. (I) Real-time PCR and (J) Western blotting were used to evaluate mRNA levels and protein levels of CDK1, respectively. **K.** The effect of pretreatment with SP600125 shows no difference from cotreatment with RO-3306. **P < 0.01 vs. respective control groups; ^##^*P* < 0.01 vs. RO-3306 group or SP600125 group; ^&&^*P* < 0.01 induction between groups.

Previous study proved that PP2A inhibitors also present an inhibition effect on PP1 (protein phosphatase 1). [13] To investigate whether these expression changes of cell cycle related genes were executed through a PP2A inhibition dependent manner, we applied siRNA targeting PP2A catalytic subunit α isoform (PP2Acα) [14] to get a specific PP2A inhibition and tested mRNA expression profile by using microarray. Knockdown of PP2Acα was firstly confirmed by real-time PCR (Figure 2A). As α and β isoforms of PP2Ac share similar coding sequence, [15] we also tested the expression of PP2Acβ. As presented in Figure 2A, expression of PP2Acβ was also repressed by PP2Acα siRNA. By using primers targeting both α and β isoforms, we confirmed the knockdown of total PP2Ac expression (Figure 2A), which resulted in the down-regulation of PP2Ac at protein level (Figure 2B).

Upon PP2Ac was knockdown, mRNA expression profile was evaluated by using microarray. The expression profiles upon treatment with PP2Acα siRNAs or PP2A inhibitors were pairwise compared. As shown in Figure 2C, Spearman's rank correlation analysis revealed that the mRNA expression values correlated significantly, suggesting the changes of expression profiles upon PP2A inhibitor treatment were mainly through inhibition on PP2A.

After cross comparison of the expressions of cell cycle related genes upon treatment with PP2A inhbitors and PP2Ac siRNAs, we identified that CDK1, the key regulator of G2/M cell cycle transition, [16] was the unique G2/M cell cycle regulator, which share the same tendence in all treated groups (Figure 2D and Figure 1E), suggesting the repression on CDK1 could be executed through PP2A inhibition dependent manner.

The down-regulation of CDK1 by PP2A inhibitor treatment was further confirmed at protein level by western blot (Figure 2E). To investigate the role of CDK1 in PANC-1 cell cycle transition and cell growth, cells were treated with RO-3306, a CDK1 inhibitor, and cell cycle assay and MTT assay were performed. As shown in Figure 2F and 2G, RO-3306 dose-dependently arrested cell cycle in G2/M phase and repressed cell growth in both dose-and time-dependent manner, suggesting CDK1 plays an important role in PANC-1 cell growth. Furthermore, pretreatment with RO-3306 impaired the cytotoxicity of PP2A inhibitors (Figure 2H), suggesting CDK1 down-regulation participated in the cytotoxicity induced by PP2A inhibitors.

To define the relationship between JNK pathway activation and CDK1 down-regulation, we pretreated cells with SP600125 and evaluated mRNA and protein levels of CDK1 upon treatment with PP2A inhibitors. As shown in Figure 2I, 2J and Supplementary Figure 2, the down-regulation of CDK1 could be reversed by pretreatment with JNK inhibitor, suggesting that down-regulation of CDK1 was through a JNK dependent pathway. Pretreatment with RO-3306 impaired the protection effect of JNK inhibitor against the cytotoxicity of PP2A inhibitors (Figure 2K), suggesting the JNK pathway dependent growth inhibition and G2/M cell cycle arrest could be executed through down-regulation of CDK1.

Thus, the PP2A/JNK/CDK1 cell signaling transition pathway dependent G2/M cell cycle arrest could be involved in the mechanism of cytotoxicity induced by PP2A inhibitors.

### PP2A inhibitors repressed CDK1 expression through activation of Sp1 transcriptional factor

**Figure 3:**
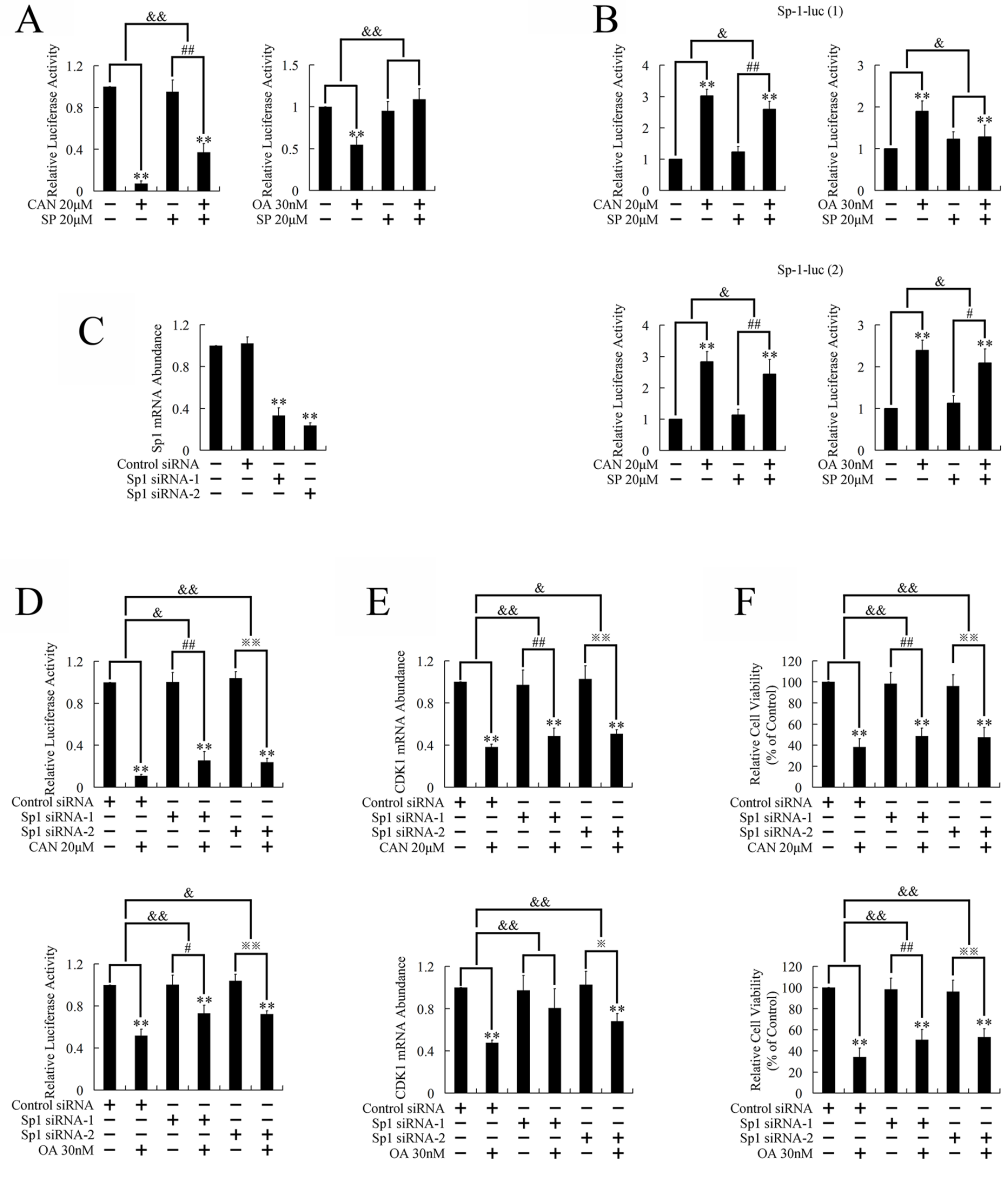
Involvement of transcriptional factor Sp1 in the JNK/CDK1 dependent cytotoxicity of PP2A inhibitors against PANC-1 cells **A.** Transcriptional activity of CDK1 promoter after treatment with PP2A inhibitors: after co-transfection with a reporter plasmid containing the CDK1 promoter and an internal plasmid; cells were pretreated with SP600125 for 3 h, followed by PP2A inhibitor treatment for 36 h; the cells were then harvested and luciferase reporter gene assays were performed. **P < 0.01 vs. respective control groups; ^##^*P* < 0.01 vs. SP600125 group; ^&&^*P* < 0.01 induction between groups. **B.** Transcriptional activation of Sp1 by PP2A inhibitors: cells were first co-transfected with a reporter plasmid containing repeating Sp1 binding sites and an internal plasmid; cells were then pretreated with SP600125, followed by PP2A inhibitor treatment for 36 h before the luciferase reporter gene assays were performed. **P < 0.01 vs. respective control groups; ^#^*P* < 0.05 and ^##^*P* < 0.01 vs. SP600125 group; ^&^*P* < 0.05 induction between groups. **C.** Real-time PCR analysis of Sp1mRNA levels after transfection with Sp1-siRNA for 24 h. **P < 0.01 vs. control group. **D.** Knockdown of Sp1 attenuated repression of the CDK1 promoter: the reporter plasmid containing CDK1 promoter and the pRL-SV40 plasmid were co-transfected 24 h after transfection with Sp1-siRNA; cells were then treated with PP2A inhibitors for 36 h, followed by luciferase assay. **E.** Knockdown of Sp1 attenuated the downregulation of CDK1 expression: after transfection with Sp1-siRNA for 24 h, cells were treated with PP2A inhibitors for a further 24 h; CDK1 expression was evaluated by Real-time PCR. **F.** Knockdown of Sp1 attenuated the inhibition of cell growth: after transfection with Sp1-siRNA for 24 h, cells were treated with PP2A inhibitors for a further 24 h; cell viability was determined by MTT assay. **P < 0.01 vs. respective control groups; ^#^*P* < 0.05 and ^##^*P* < 0.01 vs. Sp1-siRNA groups; ^&^*P* < 0.05 and ^&&^*P* < 0.01 induction between groups.

To investigate the mechanisms involved in JNK dependent down-regulation of CDK1, we cloned the CDK1 promoter [17, 18] into the pGL3-Basic luciferase reporter vector and tested its transcriptional activity after PP2A inhibition. As shown in Figure 3A, treatment with PP2A inhibitors repressed the transcriptional activity of the CDK1 promoter. Conversely, repression was attenuated by pretreatment with SP600125, the JNK inhibitor, which suggested that JNK dependent down-regulation of CDK1 was executed through repression of CDK1 transcription.

The transcriptional factor, Sp1, is a primary target of the JNK pathway; [19–24] therefore, we used luciferase reporter vectors containing the known Sp1 binding sites, GGGCGG or GGGGCGGGGC, to investigate whether Sp1 was involved in the JNK dependent down-regulation of CDK1. As shown in Figure 3B, Sp1 was activated after treatment with PP2A inhibitors; whereas pretreatment with SP600125 repressed Sp1 activation, indicating that PP2A inhibitors induced Sp1 activation through a JNK dependent pathway.

We next investigated whether repression of the CDK1 promoter, down-regulation of CDK1, and inhibition of cell growth, which were induced by PP2A inhibitors, were executed through Sp1 activation dependent manner. Real-time PCR using target specific siRNAs confirmed knockdown of Sp1 (Figure 3C). Luciferase reporter gene assays proved that interference of Sp1 attenuated repression of the CDK1 promoter (Figure 3D); real-time PCR confirmed down-regulation of CDK1 expression (Figure 3E); and MTT assays confirmed inhibition of cell growth (Figure 3F). These results suggested that PP2A inhibitors induced G2/M cell cycle arrest and repressed cell growth through the PP2A/JNK/Sp1/CDK1 pathway.

### Identification of the CDK1 promoter region which responded to the PP2A/JNK/Sp1 pathway

Activated Sp1 can either be a transcriptional activator or a repressor, depending on the stimulation, cell type, and target DNA. [25] In this study, Sp1 acted as a transcriptional activator when bound to the known Sp1 binding sites, but behaved as a transcriptional repressor when bound to the CDK1 promoter; therefore we attempted to identify the region of the CDK1 promoter that responded to Sp1 when treated with PP2A inhibitors.

**Figure 4:**
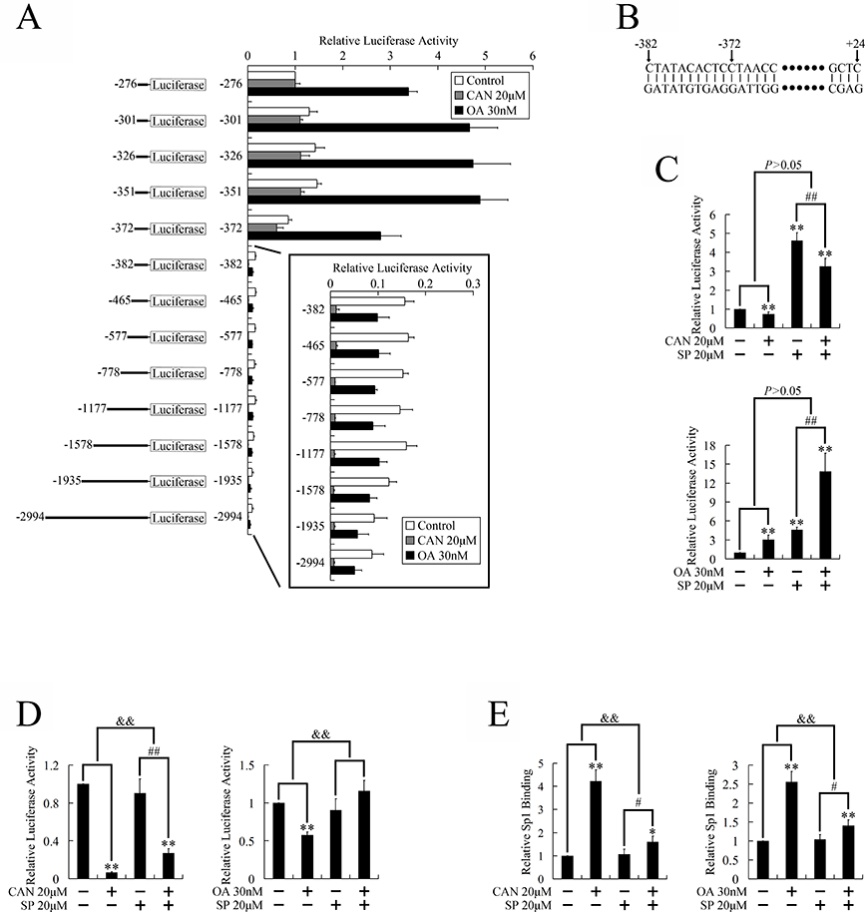
Identification of the CDK1 promoter regions which responded to PP2A inhibitors in PANC-1 cells **A.** Schematic diagram of the CDK1 promoter deletions created in the luciferase reporter construct, and the relative luciferase activities derived from the deletion constructs when transfected into PANC-1 cells. Cells were treated with PP2A inhibitors for 36 h after transfection and before the luciferase gene reporter assays were performed. The activity of the −276~+24 bp construct was given a value of 1, and the activities of the other transfections were normalized to this value. **B.** Sequence of the responsive element. **C.** JNK independent regulation of the activity of the −372~+24 bp construct; **D.** JNK dependent regulation of the activity of the −382~+24 bp construct, after treatment with PP2A inhibitors: after transfection, cells were treated with the JNK inhibitor for 3 h, and then treated with PP2A inhibitors for 36 h, followed by the luciferase gene reporter assays. **E.** JNK dependent regulation of binding between Sp1 and the responding element: cells were pretreated with SP600125 for 3 h, and then treated with PP2A inhibitors for 36 h, followed by the ChIP assays. *P < 0.05 and **P < 0.01 vs. respective control groups; ^#^*P* < 0.05 and ^##^*P* < 0.01 vs. SP600125 group; ^&^*P* < 0.05 and ^&&^*P* < 0.01 induction between groups.

The CDK1 promoter deletion constructs used for the transient transfection analysis are illustrated in Figure 4A. A significant increase of luciferase activity was observed when the base pairs between −382 and −372 were deleted, suggesting this region might contain the element that responded to PP2A inhibitor treatment (Figure 4B). The JNK inhibitor had no influence on the change in transcriptional activity induced by PP2A inhibitors when this region (−382 to −372 bp) was deleted (Figure 4C); but attenuated transcriptional repression when this region was present (Figure 4D). This suggested that PP2A inhibitors decreased CDK1 transcription through JNK pathway dependent repression of the CDK1 promoter region between −382 and −372 bp.

ChIP assays were performed to confirm the binding of Sp1 to this promoter region. As shown in Figure 4E, binding of Sp1 to this region was increased by treatment with PP2A inhibitors, but attenuated by pretreatment with the JNK inhibitor, suggesting that JNK dependent repression of this promoter region was mediated through transcriptional factor Sp1. Because no known Sp1 binding sites were found in this region, this indicated indirect binding between Sp1 and this region.

### PP2A inhibitors repressed cell growth through PP2A/JNK pathway independent and autophagy dependent up-regulation of p21

**Figure 5:**
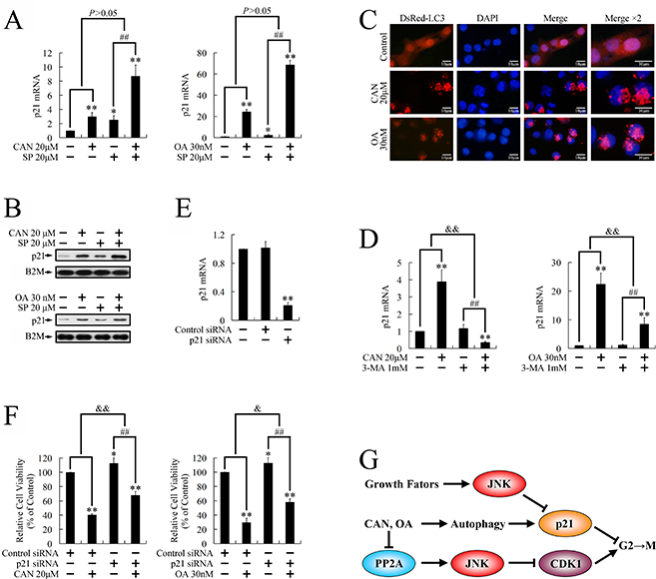
Regulation of p21 expression by cantharidin and OA in PANC-1 cells **A.** Real-time PCR and **B.** Western blotting were used to evaluate mRNA levels and protein levels of p21 after treatment with cantharidin or OA for 24 h, with or without pretreatment with SP600125. **C.** Formation of LC3 punctate after treatment with cantharidin or OA for 24 h. **D.** Real-time PCR was performed to evaluate mRNA levels of p21 after treatment with cantharidin or OA for 24 h, with or without pretreatment with 3-MA. **E.** Real-time PCR was used to confirm down-regulated mRNA levels of p21 after transfection with p21-siRNA for 24 h. **F.** After transfection with p21-siRNA for 24 h, PP2A inhibitors were added into the culture, and cells were treated cells for 48 h. Cell growth was evaluated by MTT assay. *P < 0.05, **P < 0.01 vs. respective control groups; ^##^*P* < 0.01 vs. SP600125 group; ^&^*P* < 0.05, ^&&^*P* < 0.01 induction between groups. **G.** Cell signaling pathways involved in the present study.

In our previous study, we have proved that expression of p21, the key inhibitor of CDK1, [11, 12] was upregulated upon treatment with PP2A inhibitors; [2] however, the mechanisms involved were unclear. By using microarray, we found that the down-regulation of p21 could be induced by cantharidin and OA (Figure 1E), but did not present when treated with PP2Acα siRNAs (Figure 2D), suggesting cantharidin and OA repressed p21 expression through a PP2A independent manner. To further verify whether activation of JNK pathway participated in the down-regulation of p21, we pretreated cells with SP600125 and evaluated p21 expression after treatment with cantharidin and OA. As shown in Figure 5A, 5B and Supplementary Figure 3, both the expression of p21 were up-regulated after PP2A inhibitor treatment; however, pretreatment with SP600125 had no repression on the level of up-regulation, suggesting that p21 up-regulation was not through PP2A/JNK pathway.

In our previous study, we demonstrated that autophagy induction could arrest G2/M cell cycle transition through up-regulation of p21. [26] In the present study, we tried to investigate whether this mechanism could also be involved. By using the DsRed-LC3 reporter, the increased formation of LC3 punctate was observed following the treatment with cantharidin and OA (Figure 5C and Supplementary Figure 4), suggesting these reagents induces autophagy in cancer cells. Pretreatment with 3-MA (3-Methyladenine), a classic autophagy inhibitor, attenuated or even reversed the stimulation on p21 expression by OA and cantharidin (Figure 5D and Supplementary Figure 5), suggesting the increased p21 expression was executed through an autophagy dependent manner.

To find out whether the up-regulation of p21 contributed to the cytotoxicity of cantharidin and OA, we used siRNA targeting p21 to block the p21 mediated effect on cell growth. Knockdown of p21 by siRNA was firstly confirmed by real-time PCR (Figure 5E). As shown in Figure 5F, knockdown of p21 impaired the repression on cell growth by cantharidin and OA, suggesting the autophagy dependent stimulation of p21 also participated in the cytotoxicity of cantharidin and OA.

## DISCUSSION

JNK is a kinase that is evolutionarily conserved in eukaryotes. [27] In our previous studies, the JNK pathway was found to be overactivated by treatment with PP2A inhibitors, [2, 28] which led to JNK dependent repression of cell growth. [2, 28] In this study, we focused on cell cycle regulation by the JNK pathway, and proved that PP2A inhibitors arrested the cell cycle at G2 phase through a JNK dependent manner.

The specificity of PP2A inhibitors has always been questioned for its slight inhibition effect on PP1 [13]. By using microarray, we confirmed that cantharidin and OA share extremely similar effects on expression on target genes. By using Spearman's rank correlation analysis, we found out that gene expression profiles of both cantharidin and OA were significantly correlated with PP2Ac knockdown groups. Thus, the present study proved the effects of PP2A inhibitors were mainly fulfilled through inhibition on PP2A, and both cantharidin and OA are reliable tools for investigation of PP2A. By using microarray, we identified that CDK1, the key regulator of G2/M cell cycle transition was up-regulated by PP2A inhibitors or PP2Acα targeting siRNAs. For the first time, we have found that the expression of CDK1 could be regulated by JNK pathway, and that growth inhibition of PP2A inhibitors was through the JNK/CDK1 pathway.

Sp1 is a zinc finger transcription factor that belongs to the Sp/Kruppel-like factors (KLF) family, characterized by their affinity for GC-rich promoter sequences, and is a major downstream target of the JNK pathway. [19–24] Sp1 has been shown to regulate the expression of thousands of genes involved in wide range of cellular processes, such as proliferation, growth, differentiation, and angiogenesis; [29, 30] therefore, we investigated whether the JNK dependent repression of CDK1 was mediated through Sp1. By using luciferase reporter gene assays, we demonstrated that PP2A inhibitors induced JNK upregulation of transcription at DNA sequences containing known Sp1 binding sites. This indicated that Sp1 was activated through the PP2A/JNK pathway; however, transcription of CDK1 was also repressed, suggesting that regulation of Sp1 transcription was target specific.

Post-translational modifications of Sp1 have been implicated in the regulation of Sp1. For example, phosphorylation, glycosylation, sumoylation, acetylation, and ubiquitination regulate Sp1 stability in a proteasome-dependent manner; [19, 31,–33] in addition, phosphorylation regulates the DNA binding and transcriptional activity of Sp1. [25] The following studies have proved that JNK plays an important role in the post-translational modification of Sp1: JNK activation is necessary to phosphorylate Sp1, [19, 23, 24] and to shield Sp1 from the ubiquitin-dependent degradation pathway; [19] knockdown or inhibition of JNK induced dephosphorylation resulted in the ubiquitination and degradation of Sp1; [19–23] phosphorylation of Sp1 by JNK was found to promote DNA binding [34] and transcription in some studies, [24, 35] but repress DNA binding [23] in other studies. Interestingly, okadaic acid (OA) was found to increase the level of Sp1 phosphorylation [36–38] and prevent Sp1 degradation in a JNK dependent way; [39] in addition, OA induced phosphorylation of Sp1 either promotes [38, 40] or represses [36] DNA binding, depending on the stimulation and target DNA. These data suggest that the post-translational modification of Sp1 may be involved in the transcriptional repression of CDK1 upon treatment with PP2A inhibitors.

Although no typical Sp1 binding sites were found in the DNA sequence that responded to the PP2A/JNK pathway, ChIP assays confirmed the interaction between Sp1 and the responsive element of the CDK1 promoter, which indicated indirect binding of Sp1 to this DNA region. A number of studies have demonstrated that post-translational modifications both regulate the intracellular compartmentalization of Sp1 and modulate its interactions with other transcription factors. [41–43] There are compelling data to indicate that the Sp1-dependent activation and repression of target promoters is modulated by its interactions with a repertoire of heterogeneous transcriptional regulators. A partial list of Sp1-interacting proteins includes the Mediator complex, c-Myc, Ah, Arnt, ER, AR, GATAs, p53, MEF2C, Smad2-4, Msx1, Rb, NF-κB, NF-YA, VHL, MyoD, E2F, NFAT-1, and YY1; [41] however, which transcriptional regulator participated in the regulation of Sp1 in the responsive element of the CDK1 promoter remains to be verified.

Microarray data also indicated a PP2A independent regulation on p21 by cantharidin and OA. Consisting with this result, the PP2A inhibition dependent activation of JNK had no effect on p21 expression, although the basal activity of JNK represses p21 expression, which might promote mitosis. [28, 44] This data suggested that in the normal culture environment, JNK dependent repression on p21 promoted cell growth. While in the circumstance of treatment with PP2A inhibitors, overactivation of JNK inhibited cell growth through repression on CDK1 (Figure 5G).

The PP2A/JNK independent repression on p21 was further proved to be related to autophagy induction. Autophagy is a genetically programmed, evolutionarily conserved process that degrades long-living cellular proteins and organelles, including the endoplasmic reticulum, Golgi apparatus, and mitochondria. [45] During autophagy the cytoplasmic constituents are delivered to the lysosome, forming a double-membrane vesicle, termed as autophagosome. [46] There is an increasing interest in exploring autophagy as a mechanism of action in cancer therapy. By now, 16 autophagy-related proteins (Atg proteins) have been identified to participate in autophagy. [47] LC3, the mammalian ontology of Atg8, is a credible marker for the autophagosome. [47, 48] During formation of autophagosomes, LC3 will form punctate structures within the cytoplasm that correspond to autophagic vesicles. [47]

Recent studies have explored the connection between PP2A and autophagy. PP2A can exert both a positive and negative effect on autophagy, depending on the particular step on which it acts and on its composition in subunits. [49] However, based on our present study, specific knockdown of PP2Ac did not affect the expression of p21. While treatment with cantharidin and OA stimulated p21 expression through autophagy dependent manner, suggesting the autophagy induced by cantharidin and OA might be executed through a PP2A inhibition independent manner.

In conclusion, our previous study had found that PP2A inhibitors activated the JNK pathway, induced G2/M cell cycle arrest, and inhibited growth in cancer cells. This study suggested that the PP2A/JNK/Sp1/CDK1 cell signaling pathway and autophagy/p21 pathway may be responsible for the G2/M cell cycle arrest and cytotoxicity by treatment with PP2A inhibitors in cancer cells (Figure 5G). These data also explained the bidirectional mechanism of JNK regulation in cell proliferation: the basal activity of JNK accelerated proliferation through repression of p21; whereas, overactivated JNK repressed cell growth through upregulation of CDK1.

PP2A is always considered to be a cancer repressor. [5] Previous efforts mostly focused on up-regulating PP2A to achieve an inhibition on its cancer promoting substrate kinases. [7] So it was really difficult to accept PP2A inhibitors in cancer treatment. Quite on the contrary, the dried body of mylabris, from which cantharidin is extracted, has been used as a traditional Chinese medicine for more than 2000 years and is still being used as a folk medicine. [1] Ancient Chinese applied this natural product to the treatment of cancer with the notion of “fighting fire with fire” [50] without awareness of its molecular target, PP2A. Thus, exploring the mechanism involved in the PP2A-inhibition dependent cytotoxicity, may bring new lights to cancer treatment. Our studies suggest that the PP2A/JNK pathway locates at the crossroad of determining cell fate. To maintain a normal cell function, the activity of PP2A/JNK pathway must be restricted at an appropriate level, as both repression and overactivation on this pathway could result in growth inhibition. This phenomenon fits well with the ‘Yin-Yang’ theory, which is an ancient Chinese philosophy nowadays accepted around the world. [51, 52] The principle of Yin and Yang, the balance between opposing natural forces, has been emphasised as a fundamental property of cellular growth regulation. [52] Therefore, anti-cancer approaches through activation or repression on PP2A, could both be considered as disturbing the balance between Yin and Yang. And the bidirectional JNK transducts this signal, resulting in cell growth inhibition. Our investigations bring a new cancer treatment strategy by turning a cancer-promoting kinase into an anti-cancer kinase through inhibition of PP2A, providing a whole new way to fight cancer.

## MATERIALS AND METHODS

### Cell line and cultures

The human pancreatic cancer cell line PANC-1, breast cancer cell lines, T-47D and MCF-7, and lung cancer cell lines, NCI-H292 and NCI-H1650, were purchased from the American Type Culture Collection (ATCC, VA, USA). PANC-1 cells were maintained in Dulbecco's minimum essential medium (DMEM; Gibco, NY, USA), while T-47D, MCF-7, NCI-H292 and NCI-H1650 cells were maintained in RPMI-1640 medium (Gibco), supplemented with 10% fetal calf serum (FCS; Hyclone, UT, USA), 100 units/ml penicillin, and 100 mg/ml streptomycin. The cultures were incubated at 37°C in a humidified atmosphere with 5% CO_2_. Cells were passaged every 2–3 days to obtain exponential growth.

### Reagents

Cantharidin, okadaic acid (OA), SP600125, and RO-3306 were purchased from Enzo Life Science International (PA, USA). 3-Methyladenine (3-MA) was purchased from Sigma (Sigma, MO, USA).

### Cell cycle analysis (PI staining)

Cell cycle analysis using propidium iodide (PI) was performed as previously described. [2] Prior to treatment, cells were synchronized in the cell cycle by serum starvation for 24 h. [53] After treatment, the cells were fixed in 80% ice cold ethanol, and incubated with 0.5% Triton X-100 solution containing 1 mg/ml RNase A at 37°C for 30 min. PI (Sigma) was added to a final concentration of 50 μg/ml followed by 30 min incubation in the dark. Cellular DNA content was analyzed by a fluorescence-activated cell sorter (FACS; Becton Dickinson, NJ, USA). Data were processed by ModFit LT software (Verity Software House, ME, USA).

### Cell cycle analysis (mithramycin A staining)

Cell cycle analysis with mithramycin A was performed according to the protocol established by Schmidt *et al*. [10] Briefly, the cells were incubated with 0.1% nonidet P-40 for 5 min, followed by fixation with 1% formaldehyde. Mithramycin A (Sigma) was added to the sample to a final contraction of 20 mg/ml. The green fluorescence of mithramycin A was analyzed by FACS (Becton Dickinson).

### MTT assay

Cellular growth was evaluated by MTT (3-[4, 5-dimethyltiazol-2-yl] 2, 5-diphenyl-tetrazolium bromide) assay. [54] Cells were seeded into 24-well tissue culture plates at 5 × 10^4^ cells per well. After treatment, MTT (Sigma) was added to each well to a final concentration of 0.5 mg/ml, followed by incubation at 37°C for 4 h. The medium was then removed, and 600 μl of dimethyl sulfoxide (DMSO) was added per well. As the PP2A inhibitor-treated cells became detached, the formazana in the medium was harvested by centrifugation at 10, 000 rpm for 10 min, dissolved in 200 μl of DMSO, and then returned to the original well. The absorbance of total 800 μl formazana dissolved DMSO in each well was measured at 490 nm using a microplate ELISA reader (Bio-Rad Laboratories, CA, USA). The relative cell viability was calculated as follows: relative cell viability = (mean experimental absorbance/mean control absorbance) × 100%.

### Real-time PCR

Total RNA was extracted using Trizol reagent (Invitrogen, CA, USA) according to the manufacturer's protocol. After spectrophotometric quantification, 1 μg total RNA in a final volume of 20 μl was used for reverse transcription with PrimeScript RT Reagent Kit (TaKaRa, Otsu, Shiga, Japan) according to the manufacturer's protocol. Aliquots of cDNA corresponding to equal amounts of RNA were used for quantification of mRNA by real-time PCR using the LightCycler 96 Real-time Quantitative PCR Detection System (Roche, Indianapolis, IN, USA). The reaction system (25 μl) contained the corresponding cDNA, forward and reverse primers, and SYBR Green PCR master mix (Roche). All data were analyzed using B2M gene expression as an internal standard. The specific primers were as follows: (1) p21, forward, 5′-GGACCTGTCACTGTC TTGTACC-3′, reverse, 5′-TTCCTGTGGGCGGATT AG-3′, product, 177 bp; (2) CDK1, forward, 5′-CTAGAAAGTGAA GAGGAAGGGGTT-3′, reverse, 5′-CCATGTACTGACCAG GAGGGAT-3′, product, 193 bp; (3) Sp1, forward, 5′-CAGTTGGCAGACTCTACA GC-3′, reverse, 5′-TCATGTATTCCATCAC CACC-3′, product, 345 bp; (4) PP2Ac, forward, 5′-GTTCACCAAGGAGCTGGAC CA-3′, reverse, 5′-C ATGCACATCTCCACAGACAGT AAC-3′, product, 164 bp; (5) PP2Acα, forward, 5′-CGCCAGAAGTACACGAGGAAC-3′, reverse, 5′-CGTT GGATTCTTTTGTCAGGATTT-3′, product, 240 bp; (6) PP2Acβ, forward, 5′-GGGAAACCTGCCTTTGTAT-3′, reverse, 5′-CATCATTAGTATGGCACATTTGGTC-3′, product, 156 bp; and (7) B2M, forward, 5′-TCAAGAA GGTGGTGAAGCAG-3′, reverse, 5′-AAGGTGGAGGAG TGGGTGTC-3′, product, 112 bp.

### Transfection of small interfering RNA

Target specific small interfering RNAs (siRNAs) were designed and synthesized by Genepharma (Shanghai, China). The specific sequences were as follows: (1) Control-siRNA, sense, 5′-UUCUCCGAACGUGUCACGUdTdT-3′, anti-sense, 5′-ACGUGACACGUUCGGAGAAdT dT-3′; (2) p21-siRNA, sense, 5′-GACCAUGUGGACCUGUCACd TdT-3′, anti-sense, 5′-GUGACAGGUCCACAU GGUC dTdT-3′; (3) Sp1-siRNA-1, sense, 5′-CCAGCAACAUGG GAAUUAUdTdT-3′, anti-sense, 5′-AUAAUUCCCAUG UUGC UGGdTdT-3′; (4) Sp1-siRNA-2, sense, 5′-GCCGU UGGCUAUAGCAAAUdTdT-3′, anti-sense, 5′-AUUUGC UAUAGCCAACGGCdTdT-3′; (5) PP2Acα-siRNA-1, sense, 5′-CGUGCAAGAGGUUCGAUGUdTdT-3′, anti-sense, 5′-ACAUCGAACCUCUUGCACGdTdT-3′; (6) PP2 Acα-siRNA-2, sense, 5′-GGCAGAUCUUCUGUCUA CAdTdT-3′, anti-sense, 5′-UGUAGACAGAAGAUCU GCCdTdT-3′. The transfections were performed with siRNA-Mate Transfection Reagent (Genepharma) according to the manufacturer's protocol.

### Western blot analysis

Rabbit anti-CDK1 was purchased from Cell Signaling Technology (MA, USA). Rabbit anti-p21, mouse anti-PP2Ac, and mouse anti-B2M antibodies were purchased from Santa Cruz Biotechnologies (Santa Cruz, CA, USA). Total protein was extracted using a lysis buffer containing 50 mM Tris-HCl (pH 7.4), 150 mM NaCl, 1% Triton X-100, 0.1% SDS, 1 mM EDTA, supplemented with protease inhibitors (10 mg/mL leupeptin, 10 mg/mL aprotinin, 10 mg/mL pepstatin A, and 1 mM 4-[2-aminoethyl] benzenesulfonyl fluoride). The protein extract was loaded onto an SDS-polyacrylamide gel, size-fractionated by electrophoresis, and then transferred to polyvinylidene fluoride (PVDF) membranes (Bio-Rad Laboratories). After blocking in 5% non-fat milk for 1 h, the membranes were incubated overnight with primary antibodies at 4°C. The protein expression was determined using horseradish peroxidase-conjugated antibodies followed by enhanced chemiluminescence (ECL, Millipore, St Charles, MO, USA) detection. The intensity of the bands was captured by JS-1035 image analysis scanning system (Peiqing Science & Technology, Shanghai, China). B2M was used as the internal control.

### Luciferase reporter gene plasmids

A series of deletion mutants of the human cdk1 promoter [17, 18] spanning −2994 to +24 were generated by PCR using pairs of primers bearing specific restriction sites (Table 1), and subcloned into KpnI and XhoI sites of the pGL3-basic vector. The luciferase reporter plasmid, Sp1-luc (1), which contains the sequence 5′-CGCGTGGGCGG AACTGGGCGG AGTTAGGGGCGG GA-3′, consisting of three consensus Sp1 binding sites (GGGCGG) from the SV40 promoter, was generated by subcloning the above fragment into the NheI and HindIII sites of the pGL3-basic vector, according to Sowa *et al*. [55] A further TransLucent Sp1 reporter [Sp1-Luc (2)], which contains the sequence 5′-ATTCGATCGGGGCGGGGC GAGATTAGATTCGATCGGGGCGGGGCGAG-3′, consisting of two consensus Sp1 binding sites (GGGGCGGGGC), was purchased from Panomics (CA, USA). The internal control plasmid, pRL-SV40, which contained the renilla luciferase gene was obtained from Promega (Madison, WI, USA).

**Table 1:** Sequences of the oligonucleotide pairs used by PCR to produce deletion mutants covering the CDK1 promoter *Kpn*I (GGTACC) and *XhoI* (CTCGAG) sites were added to the primers to direct subcloning, and are italicized and underlined. Positions of the DNA fragments relative to the transcription start site are indicated.

Position of DNA fragment	Sense 5′→3′	Anti-sense 5′→3′
−2994 to +24	AT*GGTACC*GATCTCCTGACAACGTGA	CTTTGAAGCCAAG*CTCGAG*CAGTTT
−1935 to +24	AC*GGTACC*AGCGGTAACTGTATCTT	CTTTGAAGCCAAG*CTCGAG*CAGTTT
−1578 to +24	ACAGCCT*GGTACC*CTAGAGCACTTC	CTTTGAAGCCAAG*CTCGAG*CAGTTT
−1177 to +24	TGGATT*GGTACC*ATGTCTAGCTCTGTTT	CTTTGAAGCCAAG*CTCGAG*CAGTTT
−778 to +24	TTTAAT*GGTACC*TAAGCCAGGCGTG	CTTTGAAGCCAAG*CTCGAG*CAGTTT
−577 to +24	GCC*GGTACC*ATAAACAAAATGTAAATA	CTTTGAAGCCAAG*CTCGAG*CAGTTT
−465 to +24	GC*GGTACC*TTGAGTTTTCCATTTCCT	CTTTGAAGCCAAG*CTCGAG*CAGTTT
−382 to +24	AA*GGTACC*TATACACTCCTAACCCTAAGT	CTTTGAAGCCAAG*CTCGAG*CAGTTT
−372 to +24	CC*GGTACC*TAACCCTAAGTATTAGAAGT	CTTTGAAGCCAAG*CTCGAG*CAGTTT
−351 to +24	TT*GGTACC*GAAAGTAATGGAATCTC	CTTTGAAGCCAAG*CTCGAG*CAGTTT
−326 to +24	ATCTCGA*GGTACC*CACAATATCACTTT	CTTTGAAGCCAAG*CTCGAG*CAGTTT
−301 to +24	AAACACAATATCACTTTTTT*GGTACC*TA	CTTTGAAGCCAAG*CTCGAG*CAGTTT
−276 to +24	TTTGAAC*GGTACC*AATGCTGGGAGAA	CTTTGAAGCCAAG*CTCGAG*CAGTTT

### Luciferase reporter gene assay

Cells were transiently co-transfected with the reporter plasmid (500 ng/well) and the internal control plasmid, pRL-SV40 plasmid (100 ng/well) for 6 h using Lipofectamine 2000 (Invitrogen), according to the manufacturer's protocol. The medium was renewed, and the treatments were initiated. After treatment, the cell lysates were subjected to the dual luciferase reporter assay (Promega) according to the manufacturer's recommendations. The luciferase activities were measured using the GloMax-20/20 luminometer (Promega). The results were expressed as relative luciferase activity (the ratio of firefly luciferase activity to renilla luciferase activity).

### ChIP assay

ChIP assays were performed using the ChIP assay kit (Upstate Biotechnology, NY, USA) as previously described. [56] Briefly, Cells (1 × 10^7^) were fixed in medium containing 1% formaldehyde for 10 min at room temperature. After cell lysis, genomic DNA was sheared into 200–1000 bp fragments using Sonics VCX130 (Sonics & Materials, CT, USA). Sheared chromatin was incubated with anti-Sp1 or negative control IgG (Upstate Biotechnology) overnight at 4°C. NaCl was added to the ChIP samples for 4 h at 65°C to reverse the cross-links. To purify the immunoprecipitated DNA the following steps were performed: RNase and proteinase K were added in order; followed by phenol-chloroform extraction; ethanol precipitation; and resuspension of the DNA in distilled water. The purified DNA was further used as a template for real-time PCR detection using an ABI Prism 7500 sequence detection system (Applied Biosystems, CA, USA). The specific primers were as follows: forward, 5′-CCTTTACATTTTTGAGGCGGTC-3′, reverse, 5′-CTCCCAGCATTGGCACAGTT-3′, product, 229 bp. Real-time PCR data were analyzed as previously described. [56]

### Microarray assay

Sample preparation and processing procedure were performed as described in detail in the Agilent GeneChip Expression Analysis Manual (Santa Clara, CA). Differentially expressed genes were screened using Agilent 44K human whole-genome oligonucleotide microarrays. The selection criterion was defined as a more than 2-fold difference in the level of expression (difference in up-regulated expression more than 2-fold, and difference in down-regulated expression less than 0.5-fold). Hierarchical clustering of samples was done by average linkage algorithm using TIGR MultiExperiment Viewer (The Institute for Genomic Research, Rockville, MD, USA).

### Autophagy assay using the DsRed-LC3 reporter

To develop an autophagy reporter, we fused the LC3 (also known as ATG8) cDNA to the C terminus of DsRed as previously described. [57] To produce stable cell lines continuously reporting autophagy activity, recombinant lentiviruses expressing the DsRed-LC3 reporter were generated and used to infect cells. The LC3 puncta were visualized with an Olympus BX51 microscope (Tokyo, Japan). DNA was counterstained using DAPI (Sigma).

### Statistical analysis

Each experiment was performed a minimum of three times. Results were expressed as the mean value ± standard deviation (SD). Statistical analysis was performed by unpaired Student's *t*-test. The correlations between microarray assays were analyzed using Spearman's rank correlation analysis. A *P* value < 0.05 was considered significant.

## SUPPLEMENTARY FIGURES



## ABBREVIATIONS

PP2A: protein phosphatase 2A
PP2Ac: PP2A catalytic subunit
OA: okadaic acid
JNK: c-Jun N-terminal kinase
CDK: cyclin-dependent kinase
CKI: cyclin-dependent kinase inhibitor
IKK: IκB kinase
ERK: extracellular signal-related kinase
PKC: protein kinase C
siRNA: small interfering RNA
PP1: protein phosphatase 1
3-MA: 3-Methyladenine
PI: propidium iodide
