# Supplementary material for: PP2A inhibitors arrest G2/M transition through JNK/Sp1-dependent down-regulation of CDK1 and autophagy-dependent up-regulation of p21
Source: Oncotarget. 2015 May 25;6(21):18469–83. doi: 10.18632/oncotarget.4063 (PMC4621904; doi:10.18632/oncotarget.4063)
Supplement: Supplementary file 1 [file oncotarget-06-18469-s001.pdf]

SUPPLEMENTARY FIGURES

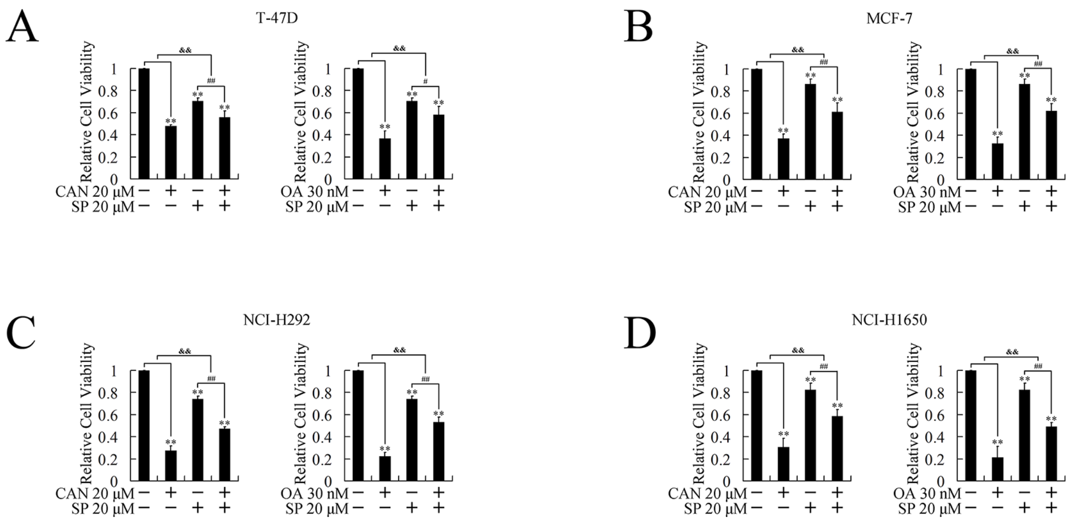

Supplementary Figure 1: JNK pathway dependent cytotoxicity of PP2A inhibitors. Breast cancer cell lines, T-47D **A.** and MCF-7 **B.** and lung cancer cell lines NCI-H292 **C.** and NCI-H1650 **D.** were pretreated with SP600125 for 3 h, followed by PP2A inhibitor treatment for 72 h. MTT assays were used to evaluate cell viability. *$P < 0.05$, **$P < 0.01$ vs. respective control groups; $^{\#}P < 0.05$, $^{\#\#}P < 0.01$ vs. SP600125 group; $^{\&}P < 0.05$, $^{\&\&}P < 0.01$ induction between groups.

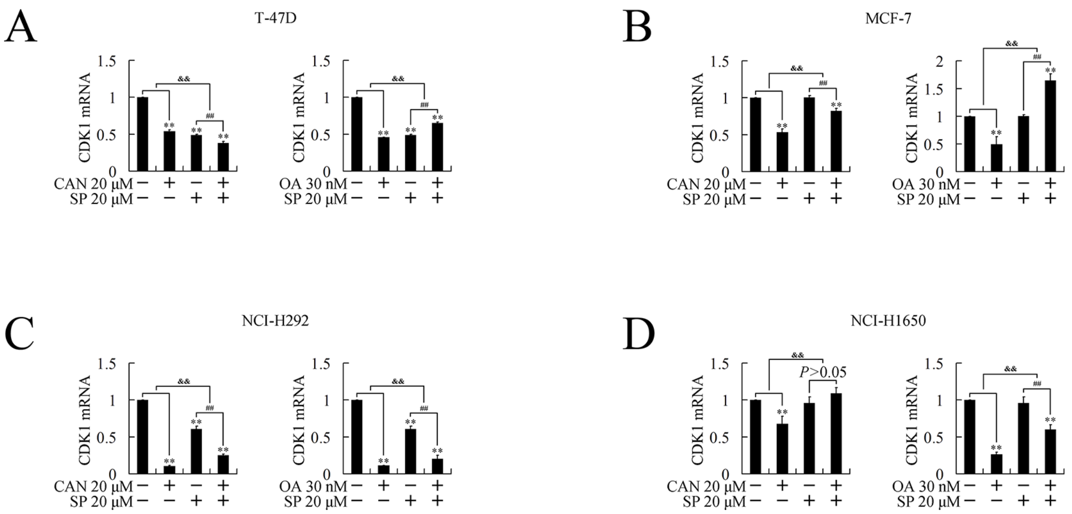

Supplementary Figure 2: JNK pathway dependent repression of CDK1 by PP2A inhibitors. After pretreatment with the JNK inhibitor, SP600125, for 3 h, PP2A inhibitors were added into the culture. T-47D **A.** MCF-7 **B.** NCI-H292 **C.** and NCI-H1650 **D.** cells were then treated for 24 h. Real-time PCR was used to evaluate mRNA levels of CDK1. *$P < 0.05$, **$P < 0.01$ vs. respective control groups; $^{\#}P < 0.05$, $^{\#\#}P < 0.01$ vs. SP600125 group; $^{\&}P < 0.05$, $^{\&\&}P < 0.01$ induction between groups.

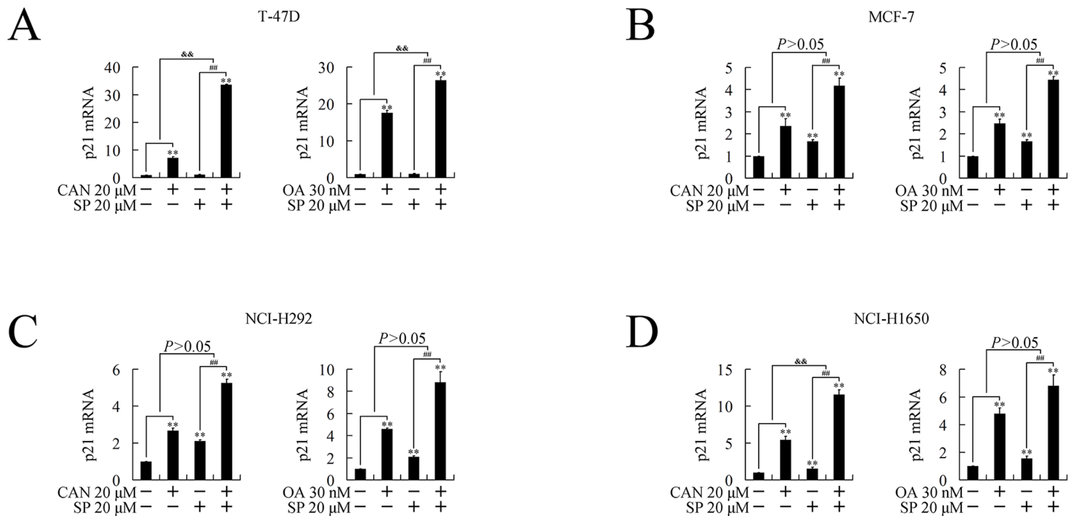

Supplementary Figure 3: JNK pathway independent up-regulation of p21 by PP2A inhibitors. After pretreatment with the JNK inhibitor, SP600125, for 3 h, PP2A inhibitors were added into the culture. T-47D **A.** MCF-7 **B.** NCI-H292 **C.** and NCI-H1650 **D.** cells were then treated for 24 h. Real-time PCR was used to evaluate mRNA levels of p21. $*P < 0.05$, $**P < 0.01$ vs. respective control groups; $^{\#}P < 0.05$, $^{\#\#}P < 0.01$ vs. SP600125 group; $^{\&}P < 0.05$, $^{\&\&}P < 0.01$ induction between groups.

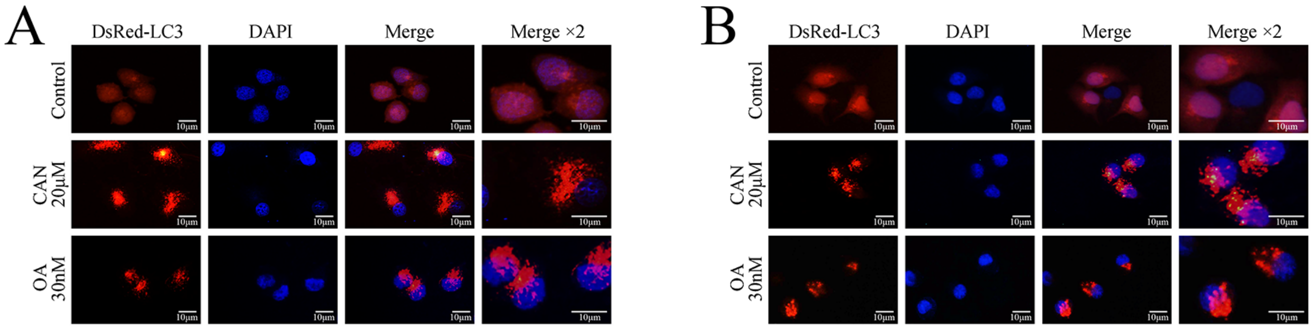

Supplementary Figure 4: Induction of autophagy by cantharidin and OA. T-47D **A.** and MCF-7 **B.** cells were treated with cantharidin or OA for 24 h. Formation of LC3 puncta were visualized with fluorescence microscope.

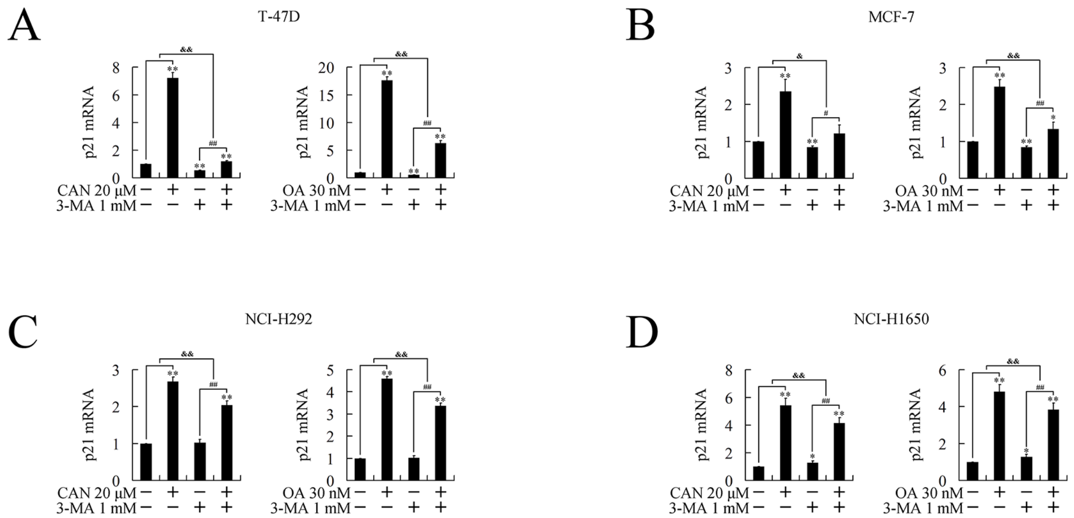

Supplementary Figure 5: Autophagic pathway dependent up-regulation of p21 by PP2A inhibitors. After pretreatment with 3-MA for 3 h, PP2A inhibitors were added into the culture. T-47D **A.** MCF-7 **B.** NCI-H292 **C.** and NCI-H1650 **D.** cells were treated for another 24 h. Real-time PCR was used to evaluate mRNA levels of p21. **P* < 0.05, ***P* < 0.01 vs. respective control groups; #*P* < 0.05, ##*P* < 0.01 vs. SP600125 group; &*P* < 0.05, &&*P* < 0.01 induction between groups.
